# Potential Adverse Drug Events with Tetrahydrocannabinol (THC) Due to Drug–Drug Interactions

**DOI:** 10.3390/jcm9040919

**Published:** 2020-03-27

**Authors:** Joshua D. Brown

**Affiliations:** Center for Drug Evaluation & Safety, Consortium for Medical Marijuana Clinical Outcomes Research, Department of Pharmaceutical Outcomes & Policy, University of Florida College of Pharmacy, Gainesville, FL 32610, USA; joshua.brown@ufl.edu

**Keywords:** medical marijuana, cannabis, THC, tetrahydrocannabinol, adverse drug events, drug–drug interactions, safety

## Abstract

Tetrahydrocannabinol (THC) is the primary psychoactive ingredient in cannabis. While the safety of THC and cannabis has been extrapolated from millennia of recreational use, medical marijuana programs have increased exposure among medically complex individuals with comorbid conditions and many co-prescribed medications. Thus, THC should be recognized as a pharmacologically complex compound with potential for drug–drug interactions and adverse drug events. This review summarizes potential adverse drug events related to THC when combined with other medications. Metabolic drug–drug interactions are primarily due to THC conversion by CYP3A4 and CYP2C9, which can be impacted by several common medications. Further, CYP2C9 polymorphisms are highly prevalent in certain racial groups (up to 35% in Caucasians) and increase the bioavailability of THC. THC also has broad interactions with drug-metabolizing enzymes and can enhance adverse effects of other medications. Pharmacodynamic interactions include neurological effects, impact on the cardiovascular system, and risk of infection. General clinical recommendations for THC use include starting with low doses and titrating to desired effects. However, many interactions may be unavoidable, dose-limiting, or a barrier to THC-based therapy. Future work and research must establish sufficient data resources to capture medical marijuana use for such studies. Meanwhile, clinicians should balance the potential risks of THC and cannabis and the lack of strong evidence of efficacy in many conditions with patient desires for alternative therapy.

## 1. Introduction

Cannabis (*Cannabis sativa*; “marijuana”) is the most commonly used illicit substance worldwide, but there is increasing interest and opportunity in employing cannabis and cannabinoids for medical purposes [1,2,3]. Legislation implemented in 33 U.S. states has not only increased the overall number of cannabis users [1], it has introduced a population of more medically complex individuals with serious chronic diseases who are now exposed to these biologically and pharmacologically active phytochemicals. The belief that cannabis is a benign medicinal plant is extrapolated from younger individuals’ recreational use, but not established for chronic use by an increasingly medically complex and older user base [4,5]. Older users in the Baby Boomer generation are, in particular, more likely to consider cannabis to be safe and to use cannabis, given generational exposure earlier in life [6,7]. As such, there is an urgent need to understand the potential, presentation, and etiology of cannabis-related adverse drug events (ADEs).

A number of components in cannabis influence, or are influenced by, drug-metabolizing enzymes (DMEs) and drug transporters which can alter the disposition of co-administered medications, i.e., pharmacokinetic drug–drug interactions (DDIs) [8,9,10,11,12,13,14,15,16,17]. Further, cannabis has significant pharmacodynamic effects, e.g., sedation and cognitive impairment, which can be potentiated with concomitant medications with similar effects or biological targets (e.g., opioids or benzodiazepines) [18,19]. Certain ADEs may also be subtle in healthy individuals but magnified in the presence of underlying medical conditions [20,21,22]. The current lack of clinical evidence related to ADEs with cannabis use creates serious patient safety concerns due to the expanding use of cannabis. Existing knowledge of cannabis-related ADEs is limited to theoretical and clinically untested hypotheses.

However, given that data sources do not yet exist to readily study DDIs with cannabinoids, there remains a need for clinical recommendations on how to best manage patients requesting cannabis treatment. Relying on existing pharmacokinetic and pharmacodynamic information, the scope of this review includes the theoretical DDIs that could occur with cannabis use. A prior review focused on cannabidiol (CBD) [23], as it is more ubiquitous in today’s regulatory environment [24] and arguably has stronger potential for metabolic DDIs. Thus, this review focused on the other primary cannabinoid, tetrahydrocannabinol (THC), which has less metabolic DDI potential but expanded pharmacodynamic interaction potential due to its effects on other physiological systems.

## 2. Approach

For this review, full prescribing information or monographs and new drug applications (NDA) were extracted from federal agency websites (e.g., U.S. FDA, Health Canada). Products included federally approved and regulated products containing THC, including Marinol and Syndros (generic name dronabinol) [25,26], which are synthetic THC prescription products; and Sativex (generic name nabiximols or THC+CBD) [9], which is a THC/CBD combination prescription product from natural extracts. CBD-only products such as Epidiolex are covered elsewhere [23]. Further, a prescription-only synthetic cannabinoid, nabilone, was excluded from this review as it is pharmacologically distinct from THC and seldom used. Prescribing information was reviewed and information on adverse events, clinical pharmacology, DDI studies, and contraindications was extracted and summarized. The review focused specifically on adverse reactions that could be attributable to DDIs or potentiated by concomitant medication use. A focused literature review was also conducted to supplement information regarding pharmacokinetic and pharmacodynamic profiles of cannabis routes of administration, ADEs reported outside of clinical trials, and prevalence of pharmacogenetic variants. DrugBank (drugbank.ca) was used as a consistent drug information resource to describe potentially interacting, enzyme substrates, and pharmacodynamic effects throughout.

## 3. Discussion

### 3.1. Regulatory Environment of THC-Containing Products

Prescription synthetic THC products have been available since the 1980s and are approved for use as antiemetics in cancer, as well as for appetite stimulation in HIV/AIDS. Dronabinol is chemically synthesized and available in capsules (Marinol^®^: 2.5, 5, and 10 mg) or an oral solution (Syndros ^®^: 5 mg/mL). Sativex^®^ (GW Pharmaceuticals, Carlsbad, CA, USA) [9], a combined Δ-9-THC and CBD product administered as a buccal spray, is indicated for spasticity and neuropathic pain in multiple sclerosis and as adjunctive analgesia for moderate to severe cancer pain. Sativex is approved in the United Kingdom, Europe, Canada, and other countries, but has not been approved for use in the U.S. A summary of these products and doses is shown in Table 1.

Cannabis is also widely available in the U.S. in individual states’ medical marijuana programs. Thirty-three (33) states allow CBD and THC use in what are deemed “comprehensive” programs, while others restrict medical cannabis products to those with low THC content [1]. Typical indications for medical cannabis include many severe conditions including inflammatory conditions (e.g., Crohn’s), chronic pain, and cancer [1]. Among state-legalized accepted uses, only chronic pain, antiemesis, multiple sclerosis, and sleep have moderate to strong evidence of cannabis effectiveness [27].

### 3.2. Potential for Adverse Drug Events and Drug–Drug Interactions

THC has broad pharmacological action, adding up to a high potential for ADEs and DDIs. These ADEs and DDIs can be pharmacokinetic in nature, i.e., generated by drug effects on other drugs absorption, metabolism, or excretion, or pharmacodynamics, wherein drugs share a common mechanism or effect. The following sections are thus divided into pharmacokinetic and pharmacodynamic groups in addition to a discussion of the molecular targets of THC.

#### 3.2.1. Molecular Targets of THC

Cannabinoid (CB) receptors make up part of the endocannabinoid system, and are associated with many of the uses of cannabis products, with roles in appetite, sleep, and pain sensations, as well as roles in the immune system, thermoregulation, and so on [28,29]. CB_1_ is implicated in the nervous system and is responsible for many of the desired effects, and neuropsychiatric adverse effects, of THC. CB_2_ receptors are primarily found in the peripheral areas of the body, with roles in anti-inflammatory and pro-inflammatory reactions and therapeutic uses of THC. THC is a partial agonist of CB receptors and, as such, leads to downregulation of these targets, with the potential to lead to tolerance and diminished efficacy over time, which should be a consideration when chronic therapy is indicated. THC has also been implicated as an inhibitor of cyclooxygenase (COX) enzymes and a potential inducer of COX-2 with prolonged exposure [30,31]. THC also has other non-CB receptor activity with somewhat unclear pharmacological effects [32]. The primary approved indication of anti-emesis and increased appetite is modulated through antagonist activity on 5-HT_3A_ receptors, and perhaps other effects on the serotonergic system [32].

#### 3.2.2. Metabolic Inhibition and Induction

THC itself is metabolized to the active metabolites 11-hydroxy-THC (11-OH-THC) and THC-COOH [33]. 11-OH-THC is considered to likely be equally, if not more, pharmacologically active as THC, and evidence also suggests that THC-COOH modulates THC effects [34]. Oxidized metabolites are formed by conversion of THC via the Cytochrome P450 enzyme system in the gastrointestinal tract and liver, including subtypes CYP2C9, CYP2C19, and CYP3A4. CYP2C9 polymorphisms can also lead to significant changes in THC bioavailability [25,26]. Polymorphisms that reduce CYP2C9 metabolic activity increased THC exposure 2- to 3-fold in clinical studies and are highly prevalent (up to 35%) in Caucasian populations, but are less prevalent in other racial groups [35,36]. Excreted metabolites of THC include the oxidized products, as well as glucuronidated compounds indicating secondary metabolism via uridine 5’-diphospho-glucuronosyltransferase (UGT) enzymes including UGT1A1/3/8/9/10 and UGT2B7. THC does not appear to be a substrate of P-glycoprotein (P-gp), but may be a substrate (and inhibitor) of other drug-transport proteins [37].

Unlike CBD, there are few modern clinical studies of THC DDIs, as product approvals are over 30 years old. However, clinical evidence is growing, including in vivo studies with certain products. In a study of Sativex (four sprays) co-administered with ketoconazole (400 mg; 5 days), a strong 3A4 inhibitor, THC bioavailability increased by 27% and 11-OH-THC by 204% (Table 2) [38]. In that scenario, 100% of the 36 health adult participants experienced an adverse event, primarily central nervous system in nature and possibly related to THC and/or 11-OH-THC toxicities. When administered with rifampicin (600 mg; 10 days), a strong CYP3A4 and CYP2C19 inducer, THC C_max_ decreased by 36% and 11-OH-THC by 87%. Omeprazole (40 mg; 6 days), a CYP2C19 inhibitor, led to no changes in THC or 11-OH-THC bioavailability [38].

**Figure 1:**
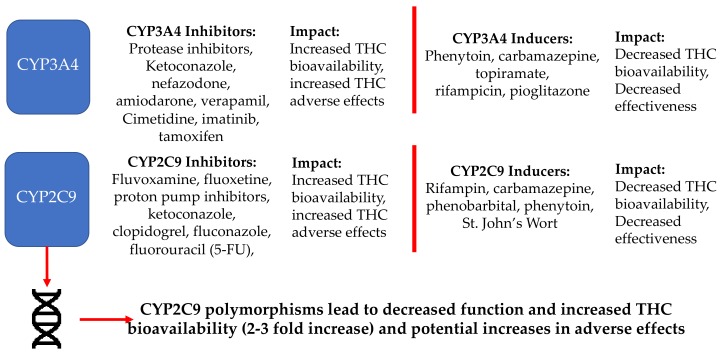
Potential pharmacokinetic drug–drug interactions involving key metabolism enzymes that convert THC to its metabolites for excretion.

Figure 1 shows common medications that are inhibitors or inducers of enzymes relevant to THC’s metabolism and potential effects. Overall, medications that interact with THC metabolism may influence the degree of pharmacological effects, including ADEs with THC discussed below. Initial THC doses should be low and titrated to desired effects when paired with medications that inhibit metabolic enzymes. Conversely, higher THC doses may be needed when inducers are present. Care should also be taken when discontinuing medications and doses should be titrated accordingly.

**Figure 2:**
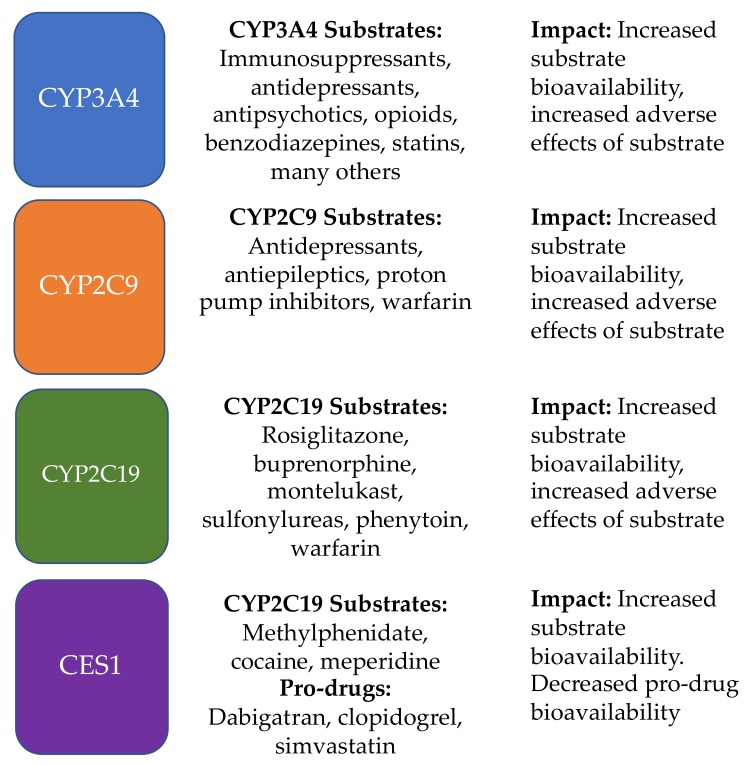
Enzyme targets and example medications that could be affected by THC if co-administered.

THC has been shown to have broad inhibitory effects on CYP450 enzymes including CYP3A, CYP2D6, CYP2C9, CYP2C19, CYP2A6, CYP2B6, CYP1A1/2, and CYP2J2 [39]. In addition, new evidence suggests that cannabinoids, including THC, have strong inhibitory effects on carboxylesterase 1 (CES1), which is important in the metabolism of many medications [39,40,41]. Overall, THC is less implicated in DDIs compared to CBD, but is still likely to contribute to this risk at clinically relevant concentrations. More importantly, whereas CBD is frequently used as a standalone cannabinoid, THC (except for prescription products) is practically always combined with other cannabinoids and the synergistic effects increase the probability and magnitude of potential DDIs. Figure 2 shows common medications that are metabolized by enzymes which THC inhibits, and potential clinical impacts.

#### 3.2.3. Synergistic Pharmacodynamic Effects

**Figure 3:**
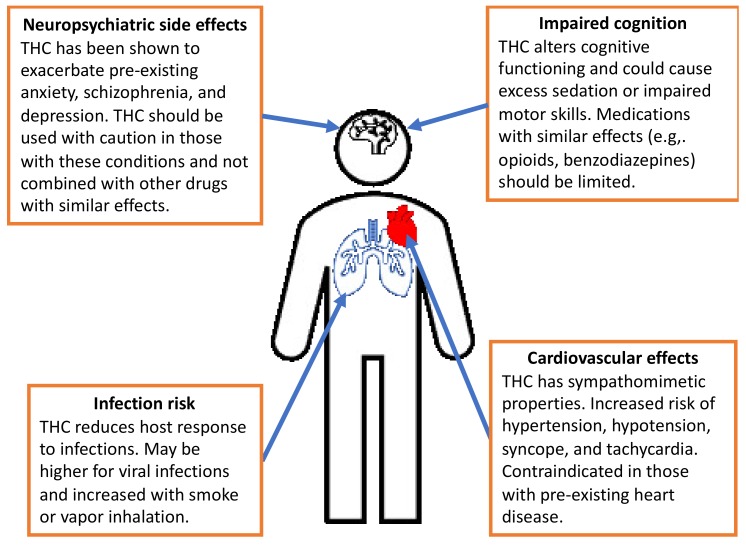
Representation of main adverse effects of tetrahydrocannabinol use that can be potentiated by other medications.

Variation in the pharmacodynamic effects of THC can be generated due to variation in the specific dispensary, manufacturer, farm, and batch. Additional patient-level variation is introduced based on physiology, route of administration, and administration practices. Further, smoking and vaping of products introduces the chance of oxidizing many compounds in cannabis. These concepts were discussed in detail in our prior review [23]. Inhaled products may also increase the risk of infection, particularly respiratory infections such as pneumonia, similarly to the effect seen with cigarette smoking [42]. These main effects are summarized in Figure 3. It is worth noting again that neuropsychiatric effects are often dose-limiting, and are also the primary reasons for discontinuation beyond lack of efficacy [38,43].

#### 3.2.4. Neuropsychiatric Side Effects

Key warnings around cannabis and THC focus on the neuropsychiatric side effects, which are dose-limiting and a primary cause of discontinuation. Dronabinol specifically was shown to exacerbate mania, depression, and schizophrenia in clinical trials of dronabinol. In a recent meta-analysis, cannabinoids versus comparator groups had nearly three times the odds of psychiatric or nervous system disorder side effects when studied as a combined endpoint, while individual symptoms (e.g., anxiety, depression) were not statistically significant [44]. In a recent study of medical use, cannabis was shown to reduce neuropsychiatric conditions (i.e., depression, anxiety, and stress) in the short term, while exacerbating depression over long-term use [45]. Nevertheless, FDA labeling recommends screening patients prior to initiating therapy with THC, and this is a recommendation that should be extrapolated to initiation of medical cannabis regimens [25,26]. In addition, THC has the potential for development of dependency and should be used in caution in individuals with previous or current substance use disorders, including those involving nicotine, alcohol, opioids, or other illicit drugs. While perhaps sought out in recreational use, clinical trial populations also reported disorientation, dissociation, euphoria, and hallucination, which may be detrimental side effects in medically severe patients, particularly older adults.

It has been noted in prescribing information for recently approved products that psychoactive medications carry an increased risk of suicidal behavior and ideation [46]. Other common medications may also increase the risk of depression, such as antihypertensives, antidepressants, and opioids, in addition to depression being common in those with chronic conditions [47,48]. Similar to recommendations for CBD [23], but perhaps needing to be more emphasized for THC-containing products, medical cannabis should be used in caution in persons with depression or who use other medications that carry this risk. For those already being treated with antipsychotics or antidepressants, selecting those without DDI potential first has been suggested [49].

#### 3.2.5. Cognitive Adverse Reactions

THC can cause cognitive impairment and an altered mental state. Reported ADEs in clinical trials included amnesia, impaired balance, disturbed attention, dizziness, lethargy, and somnolence. Co-administration with other medications with similar effects will likely potentiate lethargic and sedative effects and may lead to excessive sedation, interruption in daily activities or work, and create a public health hazard, e.g., driving under the influence of cannabis [50,51]. Co-administration should be avoided and patients counseled to avoid hazardous activities while being treated. However, cognitive effects should be balanced against patient needs, as these are often the desired effects sought with medical cannabis treatment. Sedation, euphoria, and dissociative effects have influences on severe pain and anxiety in serious diseases, particularly cancer. In fact, a recent study showed that cancer patients are more likely to use THC versus non-cancer patients. Thus, while cognitive ADEs should be avoided in general, a risk–benefit assessment is needed for this and all other serious side effects.

#### 3.2.6. Cardiovascular Side Effects

Sativex carries a contraindication in its international product labeling for any users with pre-existing cardiovascular disease, which is likely attributable solely to THC’s sympathomimetic properties [9]. Thus, for the nearly half of U.S. adults with some form of heart disease, and for older patients with severe comorbidities in particular, THC should be reconsidered. Dronabinol alone has been associated with hypotension, hypertension, syncope, and tachycardia [25,26]. In older adults, there may be the potential to adversely lower blood pressure, more so in individuals who are treated with antihypertensives or other drugs that cause hypotension or syncope (e.g., medications with anticholinergic effects [52]), which could lead to falls and negative sequelae.

#### 3.2.7. Infection Risk

One of the primary foci of cannabis research is its anti-inflammatory properties, which are mediated through the endocannabinoid system and COX enzymes [30,32]. However, evidence suggest that, like many other anti-inflammatory agents, this is a trade-off with protective host immune response. THC alone has been shown to reduce T-helper cell response, reduce fever response, reduce in pro-inflammatory cytokines, and reduce overall host mortality in murine models [53,54,55,56]. Little evidence is available for the same effect in humans specifically for THC. However, as a previous review showed, infections were more common with CBD alone as well [23]. Caution should be considered in patients taking immunosuppressant medications, and clinicians may also consider reinforcement of vaccination regimens prior to treatment in high-risk patients. This recommendation mirrors similar recommendations prior to initiating other immune-modulating medications.

## 4. Conclusions

Anecdotal evidence suggests that medical cannabis is a benign product with the potential to treat a myriad of conditions with few side effects. However, as the current evidence basis shows, there is little evidence supporting its efficacy in many conditions and many additional safety concerns that should be considered [44]. This review considered the pharmacology of THC, with the caveat that THC will not be used alone in medical marijuana programs. Nevertheless, THC exhibits specific ADE potential that is dose-limiting and distinct from other cannabinoid preparations, and may limit any perceived benefits. These effects primarily include the risk of neuropsychiatric events, cardiovascular effects, impaired cognition and sedation, and infection risk.

Further, THC’s effects can be enhanced or limited by many other common medications, and THC itself can increase toxicity of other medications by limiting their metabolic pathways. These risks are more pronounced in the medically complex patients towards whom medical marijuana programs are tailored. Increased awareness of these effects by clinicians is needed to mitigate risks and actions such as starting with low-dose therapy, slow titration, or avoiding THC-containing products altogether, i.e., initiating CBD-only treatment. These considerations must, nevertheless, also balance patient desires for alternative therapies and the underlying indication for medical cannabis therapy. Recognizing THC and other cannabinoids as medical substances may shift the perception of these compounds towards being considered in equal regards to prescription products in terms of risks and benefits. Moving forward, clinicians can adopt the old adage of “start low and go slow” when it comes to dosing THC and other cannabinoids with the goal of reducing ADEs while providing necessary and desired treatments.

## Figures and Tables

**Table 1:** Product information for cannabis-derived pharmaceutical products that contain tetrahydrocannabinol (THC).

Product (Approval Date)	Active Ingredient(s)	Dosage Form	Route	Recommended Dose	Indication(s)
SATIVEX ^a^ (2011–12)	Delta-9-THC and cannabidiol	Solution, spray	Buccal Spray	Titrated up to 12 sprays per day (patient median is 4–8 sprays). 2.7 mg THC and 2.5 mg CBD per spray.	Adjunctive treatment of spasticity and neuropathic pain in MS
					Adjunctive analgesic for moderate to severe pain in advanced cancer
MARINOL (1985)	Dronabinol ^b^	Capsules	Oral	2.5 mg 2× daily; max 5 mg 2× daily	Anorexia associated with AIDS
				5 mg/m^2^ 4–6× daily; max 15 mg/m^2^ 4–6× daily	Nausea and vomiting with chemotherapy in patients for whom conventional treatment failed
SYNDROS (1985)	Dronabinol ^b^	Solution	Oral	2.1 mg 2× daily; max 8.4 mg daily	Anorexia associated with AIDS
				4.2 mg/m^2^ 4–6× per day; max 12.6 mg/m^2^ 4–6× per day	Nausea and vomiting with chemotherapy in patients for whom conventional treatment failed

^a^ Sativex is not approved in the United States, but was approved in most other countries between 2011 and 2012. ^b^ Dronabinol is a synthetic form of THC not extracted from cannabis. THC = tetrahydrocannabinol; MS = multiple sclerosis.

**Table 2:** In vivo pharmacokinetic drug–drug interaction study of Sativex [38].

Product	Population Studied	Interacting Drugs	Results
THC+CBD mucosal spray (Sativex), 4 sprays	Healthy Adults (*n* = 36)	Rifampicin 600 mg (CYP3A, CYP2C19 inducer)	THC: 36% decrease 11-OH-THC: 87% decrease
		Ketoconazole 400 mg (CYP3A inhibitor)	THC: 27% increase 11-OH-THC: 204% increase
		Omeprazole 40 mg (CYP2C19 inhibitor)	No change in THC or 11-OH-THC

All participants in the study reported a serious adverse effect when THC was combined with ketoconazole, primarily neuropsychiatric in nature.
